# Supplementary material for: From sequence to enzyme mechanism using multi-label machine learning
Source: BMC Bioinformatics. 2014 May 19;15:150. doi: 10.1186/1471-2105-15-150 (PMC4229970; doi:10.1186/1471-2105-15-150)
Supplement: Additional file 2 — Java code of ml2db. Additional file ml2db_code.tar.gz contains the Java source code to run the multi-label machine learning experiments and save the results to database. The code’s Javadoc is included. [file 1471-2105-15-150-S2.zip › additional file 2/ml2db/utils/doc/index-files/index-20.html]

T-Index


JavaScript is disabled on your browser.


- Overview
- Package
- Class
- Use
- Tree
- Deprecated
- Index
- Help

- Prev Letter
- Next Letter

- Frames
- No Frames

- All Classes

A B C D E F G H I J K L M N O P Q R S T U V W X Y 


## T

Table - Class in uk.ac.standrews.utils.main.database
:   An SQL database table definition (table name + columns)

Table(String, Vector<TableColumn>, DbManager) - Constructor for class uk.ac.standrews.utils.main.database.Table
:   Construct a table for writing to: the columns definitions (name, data type) must be known.

Table(String, DbManager) - Constructor for class uk.ac.standrews.utils.main.database.Table
:   Construct a table read-only.

TABLE1 - Static variable in class uk.ac.standrews.utils.test.database.TableCreatorTest


TableColumn - Class in uk.ac.standrews.utils.main.database
:   A column object contains all the information to create, fill and delete a
    database table column

TableColumn(String, String, boolean) - Constructor for class uk.ac.standrews.utils.main.database.TableColumn
:   Constructor 2: takes the column name, data type and primary key status

TableCreator - Class in uk.ac.standrews.utils.main.database
:   Class to create a table object in the database.

TableCreator(Table) - Constructor for class uk.ac.standrews.utils.main.database.TableCreator


TableCreatorTest - Class in uk.ac.standrews.utils.test.database
:   Class

TableCreatorTest() - Constructor for class uk.ac.standrews.utils.test.database.TableCreatorTest


tableExistsInDb(String) - Method in class uk.ac.standrews.utils.main.database.DbReader
:   Checks if a table exists in the database

TableManager - Class in uk.ac.standrews.utils.main.database
:   Class to create/read/write to a database table.

TableManager(Table) - Constructor for class uk.ac.standrews.utils.main.database.TableManager
:   Constructor to create a manager to create, read and write a table.

TableMap - Class in uk.ac.standrews.utils.main.maputils
:   A table map is a map containing relations [key > list of values] [value >
    list of keys]

TableMap() - Constructor for class uk.ac.standrews.utils.main.maputils.TableMap
:   Initialises the object with an empty 'one to many' map.

TableMap(Vector<String>, Vector<String>) - Constructor for class uk.ac.standrews.utils.main.maputils.TableMap
:   Initialise with a list of key-value pairs

TableMapTest - Class in uk.ac.standrews.utils.test.maputils
:   Class

TableMapTest() - Constructor for class uk.ac.standrews.utils.test.maputils.TableMapTest


TableReader - Class in uk.ac.standrews.utils.main.database
:   Class

TableReader(Table) - Constructor for class uk.ac.standrews.utils.main.database.TableReader


TableReaderTest - Class in uk.ac.standrews.utils.test.database
:   Class

TableReaderTest() - Constructor for class uk.ac.standrews.utils.test.database.TableReaderTest


TableRow - Class in uk.ac.standrews.utils.main.database
:   Contains the data for a generic row (a list of [table column, value] entries)
    to be written to a database table.

TableRow(Table) - Constructor for class uk.ac.standrews.utils.main.database.TableRow


TableRow(Table, TreeMap<String, String>) - Constructor for class uk.ac.standrews.utils.main.database.TableRow


TableRow(Table, Vector<String>, Vector<String>) - Constructor for class uk.ac.standrews.utils.main.database.TableRow


TableRowTest - Class in uk.ac.standrews.utils.test.database
:   Class

TableRowTest() - Constructor for class uk.ac.standrews.utils.test.database.TableRowTest


TableTest - Class in uk.ac.standrews.utils.test.database
:   Class

TableTest() - Constructor for class uk.ac.standrews.utils.test.database.TableTest


TableWriter - Class in uk.ac.standrews.utils.main.database
:   A TableWriter object contains all the information to create, fill and delete
    a database table.

TableWriter(Table) - Constructor for class uk.ac.standrews.utils.main.database.TableWriter
:   Constructor 2: takes the table name and a list of fields

TableWriterTest - Class in uk.ac.standrews.utils.test.database
:   Class

TableWriterTest() - Constructor for class uk.ac.standrews.utils.test.database.TableWriterTest


tearDown() - Method in class uk.ac.standrews.utils.test.database.DbConnPropsTest


tearDown() - Method in class uk.ac.standrews.utils.test.database.DbReaderTest


TEST\_DB\_CONN\_PATH - Static variable in class uk.ac.standrews.utils.main.PathUtils


testAddAll() - Method in class uk.ac.standrews.utils.test.maputils.OneToManyMapTest


testAddChildElement() - Method in class uk.ac.standrews.utils.test.XmlNodeTest


testAddKeyValue() - Method in class uk.ac.standrews.utils.test.maputils.OneToManyMapTest


testAddSubset() - Method in class uk.ac.standrews.utils.test.setutils.SetTest


testAddValue() - Method in class uk.ac.standrews.utils.test.maputils.TableMapTest


testAddValueToValues() - Method in class uk.ac.standrews.utils.test.maputils.OneToManyMapTest


testCanWriteParent() - Method in class uk.ac.standrews.utils.test.FileUtilsTest


testCheckRow() - Method in class uk.ac.standrews.utils.test.database.TableRowTest


testCheckRowFields() - Method in class uk.ac.standrews.utils.test.database.TableRowTest


testClass() - Method in class uk.ac.standrews.utils.test.UtilsTest
:   public void testBigDecimal(){ this.testBigDecimalGeneric(0.0, 1.0, 0.1);
    this.testBigDecimalGeneric(0.0, 1.0, 0.2);
    this.testBigDecimalGeneric(0.0, 1.0, 0.3); }
    public void testDoubleIncrements1(){
    this.testDoubleIncrementsBigDecimal(0.0, 10.0, 0.1);
    this.testDoubleIncrementsBigDecimal(0.0, 1.0, 0.1);
    this.testDoubleIncrementsBigDecimal(0.0, 1.0, 0.2);
    this.testDoubleIncrementsBigDecimal(0.0, 1.0, 0.3);
    // is not well rounded this.testDoubleIncrementsBigDecimal(0.0, 1.7,
    0.3);
    // increment is null Vector increments =
    this.testDoubleIncrementsBigDecimal(0.0, 10.0, 0.0);
    //assertNull(increments); }
    public Vector testDoubleIncrementsBigDecimal(Double min,
    Double max, Double percOfIncrement){ TreeSet basicIncrements
    = Utils.getDoubleIncrementsBigDecimal(min, max, percOfIncrement);
    if(basicIncrements != null){ Vector increments = new
    Vector(basicIncrements); Double range = max - min; Double
    increment = range \* percOfIncrement;
    if(increment > 0 || increment < 0){ System.out.println("min: " + min +
    ", max: " + max + ", increment: " + increment + " " +
    increments.toString());
    Double noOfincrements = (range / increment) + 1; int numberOfIncrements =
    noOfincrements.intValue();
    //assertEquals(numberOfIncrements, increments.size()); for(int i=0; i <
    numberOfIncrements; i++ ){ Double value = min + (i \* increment);
    //assertEquals(value, (Double) increments.get(i)); }
    }else{ //assertEquals(null, increments); } return increments; }else{
    return null; } }
    public void testBigDecimalGeneric(Double minDouble, Double maxDouble,
    Double percentOfIncrementDouble){ BigDecimal min =
    BigDecimal.valueOf(minDouble); BigDecimal max =
    BigDecimal.valueOf(maxDouble); BigDecimal percentOfIncrement =
    BigDecimal.valueOf(percentOfIncrementDouble);
    Vector intervals = new Vector(); BigDecimal range
    = max.subtract(min);
    System.out.println("UtilsTest BigDecimal: " + min + " " + max + " " +
    percentOfIncrement + " " + range); System.out.println("zero " +
    BigDecimal.ZERO); assertEquals(1, range.compareTo(BigDecimal.ZERO));
    BigDecimal increment = range.multiply(percentOfIncrement);
    System.out.println("range.divide(increment, RoundingMode.DOWN) " +
    range.divide(increment, RoundingMode.DOWN)); BigDecimal value = min;
    value = value.add(increment); System.out.println("value.add(increment) "
    + value); System.out.println("value.add(increment).ROUND\_DOWN " +
    value.add(increment).ROUND\_DOWN); System.out.println("max.ROUND\_DOWN " +
    max.ROUND\_DOWN); System.out.println("---------------------------------");
    BigDecimal one0 = BigDecimal.valueOf(1.0); BigDecimal one1 =
    BigDecimal.valueOf(1.0); BigDecimal range0 = one0.subtract(one1);
    assertTrue(range0.compareTo(BigDecimal.ZERO) == 0);
    }

testColumnExistsInTable() - Method in class uk.ac.standrews.utils.test.database.TableReaderTest


testConnection() - Method in class uk.ac.standrews.utils.test.database.DbManagerTest


testCountValues() - Method in class uk.ac.standrews.utils.test.CollectionUtilsTest


testCreateTable() - Method in class uk.ac.standrews.utils.test.database.TableCreatorTest


testDateConverter() - Method in class uk.ac.standrews.utils.test.TimeUtilsTest


testDepthFirstSearchOfParent() - Method in class uk.ac.standrews.utils.test.XmlSearcherTest


testDoubleFormat() - Method in class uk.ac.standrews.utils.test.NumberUtilsTest


testDoubleIncrements2() - Method in class uk.ac.standrews.utils.test.UtilsTest


testDropTable() - Method in class uk.ac.standrews.utils.test.database.TableCreatorTest


testElapsedTime() - Method in class uk.ac.standrews.utils.test.TimeUtilsTest


testExtractDouble() - Method in class uk.ac.standrews.utils.test.stats.UniformRandomUtilsTest


testExtractInteger() - Method in class uk.ac.standrews.utils.test.stats.UniformRandomUtilsTest


testExtractUniformRandomMapEntry() - Method in class uk.ac.standrews.utils.test.stats.UniformRandomUtilsTest


testFillMap() - Method in class uk.ac.standrews.utils.test.maputils.TableMapTest


testFilterListsByRegExp() - Method in class uk.ac.standrews.utils.test.RegExpUtilsTest


testFormatPercentage() - Method in class uk.ac.standrews.utils.test.NumberUtilsTest


testFrequenciesToPercentOfFrequency() - Method in class uk.ac.standrews.utils.test.stats.StatUtilsTest


testGetChildByTag() - Method in class uk.ac.standrews.utils.test.XmlSearcherTest


testGetColumns() - Method in class uk.ac.standrews.utils.test.database.TableTest


testGetConnection() - Method in class uk.ac.standrews.utils.test.database.DbConnPropsTest


testGetConnectionLog() - Method in class uk.ac.standrews.utils.test.database.DbConnPropsTest


testGetDatabaseName() - Method in class uk.ac.standrews.utils.test.database.DbConnPropsTest


testGetDatabaseType() - Method in class uk.ac.standrews.utils.test.database.DbConnPropsTest


testGetDriverName() - Method in class uk.ac.standrews.utils.test.database.DbConnPropsTest


testGetFileNameFromDirectory() - Method in class uk.ac.standrews.utils.test.FileUtilsTest


testGetFileNameFromDirectory2() - Method in class uk.ac.standrews.utils.test.FileUtilsTest


testGetHighestValuedKey() - Method in class uk.ac.standrews.utils.test.maputils.MapUtilsTest


testGetHighestValuedKeyRandomly() - Method in class uk.ac.standrews.utils.test.maputils.MapUtilsTest


testGetHost() - Method in class uk.ac.standrews.utils.test.database.DbConnPropsTest


testGetInsertSql() - Method in class uk.ac.standrews.utils.test.database.TableWriterTest


testGetInstance() - Method in class uk.ac.standrews.utils.test.stats.UniformRandomSingletonTest


testGetKeys() - Method in class uk.ac.standrews.utils.test.maputils.TableMapTest


testGetKeysOrValuesSet() - Method in class uk.ac.standrews.utils.test.maputils.OneToManyMapTest


testGetKeysVectorValuesVector() - Method in class uk.ac.standrews.utils.test.maputils.OneToManyMapTest


testGetKeyValueNumber() - Method in class uk.ac.standrews.utils.test.maputils.TableMapTest


testGetListOfDbTables() - Method in class uk.ac.standrews.utils.test.database.DbReaderTest


testGetLoadInfileSql() - Method in class uk.ac.standrews.utils.test.database.TableWriterTest


testGetMatchesBoundaries() - Static method in class uk.ac.standrews.utils.test.RegExpUtilsTest


testGetNonEmptyKeysOrValues() - Method in class uk.ac.standrews.utils.test.maputils.OneToManyMapTest


testGetParetoFrequencies() - Method in class uk.ac.standrews.utils.test.stats.TruncatedParetoTest


testGetReaderFromURL() - Method in class uk.ac.standrews.utils.test.WebUtilsTest


testGetSelectWhereEqualSql() - Method in class uk.ac.standrews.utils.test.database.SqlUtilsTest


testGetSqlTimestamp() - Method in class uk.ac.standrews.utils.test.TimeUtilsTest


testGetSqlToCreateTable() - Method in class uk.ac.standrews.utils.test.database.TableCreatorTest


testGetSqlToDropTable() - Method in class uk.ac.standrews.utils.test.database.DbCreatorTest


testGetStatement() - Method in class uk.ac.standrews.utils.test.database.DbConnPropsTest


testGetTableFields() - Method in class uk.ac.standrews.utils.test.database.TableReaderTest


testGetTableName() - Method in class uk.ac.standrews.utils.test.database.TableCreatorTest


testGetTableName() - Method in class uk.ac.standrews.utils.test.database.TableTest


testGetTidyDOM() - Method in class uk.ac.standrews.utils.test.XmlUtilsTest


testGetUniformRandomSubmap() - Method in class uk.ac.standrews.utils.test.stats.UniformRandomUtilsTest


testGetValues() - Method in class uk.ac.standrews.utils.test.maputils.TableMapTest


testGetValuesForAKey() - Method in class uk.ac.standrews.utils.test.maputils.OneToManyMapTest


testGetValuesForAKey() - Method in class uk.ac.standrews.utils.test.maputils.TableMapTest


testGetXmlFromHTML() - Method in class uk.ac.standrews.utils.test.XmlUtilsTest


testHasKey() - Method in class uk.ac.standrews.utils.test.database.TableTest


testHistogram() - Method in class uk.ac.standrews.utils.test.stats.StatUtilsTest


testHumanReadableTimestamp() - Method in class uk.ac.standrews.utils.test.TimeUtilsTest


testIncrementsUtil() - Method in class uk.ac.standrews.utils.test.UtilsTest


testIndexedMap() - Method in class uk.ac.standrews.utils.test.maputils.MapUtilsTest


testInsert() - Method in class uk.ac.standrews.utils.test.database.DbWriterTest


testIntegerIncrements(Integer, Integer, Double) - Method in class uk.ac.standrews.utils.test.UtilsTest


testIsDash() - Method in class uk.ac.standrews.utils.test.StringUtilsTest


testIsSubset() - Method in class uk.ac.standrews.utils.test.setutils.SetTest


testJavaReflectionGetFields() - Method in class uk.ac.standrews.utils.test.ReflectionUtilsTest
:   only works for public fields

testJavaReflectionMethods() - Method in class uk.ac.standrews.utils.test.ReflectionUtilsTest


testListIsNullOrEmpty() - Method in class uk.ac.standrews.utils.test.ListUtilsTest


testManager() - Method in class uk.ac.standrews.utils.test.setutils.SupersetsManagerTest


testMapIsFilled() - Method in class uk.ac.standrews.utils.test.maputils.MapOfMapsTest


testMapIsFilled() - Method in class uk.ac.standrews.utils.test.maputils.OneToManyMapTest


testMapToString() - Method in class uk.ac.standrews.utils.test.maputils.MapUtilsTest


testMd5Hash() - Method in class uk.ac.standrews.utils.test.WebUtilsTest


testMillisecTimeStamp() - Method in class uk.ac.standrews.utils.test.TimeUtilsTest


testMultilineMatch() - Method in class uk.ac.standrews.utils.test.RegExpUtilsTest


testNanosecStamp() - Method in class uk.ac.standrews.utils.test.TimeUtilsTest


testNextInt() - Method in class uk.ac.standrews.utils.test.stats.TruncatedParetoTest


testNodeHeadToString() - Method in class uk.ac.standrews.utils.test.XmlNodeTest


testObjectIncrements() - Method in class uk.ac.standrews.utils.test.UtilsTest


testPareto() - Method in class uk.ac.standrews.utils.test.stats.ParetoTest


testPareto() - Method in class uk.ac.standrews.utils.test.stats.TruncatedParetoTest


testParetoOriginal() - Method in class uk.ac.standrews.utils.test.stats.StatUtilsTest


testPerformance() - Method in class uk.ac.standrews.utils.test.stats.PseudoTruncatedParetoTest


testPickInteger() - Method in class uk.ac.standrews.utils.test.stats.RandomUtilsTest


testProbabilityDensityFunction() - Method in class uk.ac.standrews.utils.test.stats.StatUtilsTest
:   public void testCumulativeDistributionFunction(){ ParetoDistribution
    pareto = new ParetoDistribution();
    System.out.println(pareto.getPareto().cdf(1.0));
    System.out.println(pareto.getPareto().cdf(2.0));
    System.out.println(pareto.getPareto().cdf(2.5));
    System.out.println(pareto.getPareto().cdf(3.0));
    System.out.println(pareto.getPareto().cdf(10.5));
    System.out.println(pareto.getPareto().cdf(100.5));
    System.out.println("-------");
    }

testPseudoTruncatedPareto() - Method in class uk.ac.standrews.utils.test.stats.PseudoTruncatedParetoTest


testQuoteIfNeeded() - Method in class uk.ac.standrews.utils.test.StringUtilsTest


testRandomDoubleNumber() - Method in class uk.ac.standrews.utils.test.stats.RandomUtilsTest


testRandomIntNumber() - Method in class uk.ac.standrews.utils.test.stats.RandomUtilsTest


testRandomSubmap() - Method in class uk.ac.standrews.utils.test.stats.RandomUtilsTest


testRemoveKeyValue1() - Method in class uk.ac.standrews.utils.test.maputils.OneToManyMapTest


testRemoveValue() - Method in class uk.ac.standrews.utils.test.maputils.TableMapTest


testRemoveValueEverywhere() - Method in class uk.ac.standrews.utils.test.maputils.OneToManyMapTest


testRightChop() - Method in class uk.ac.standrews.utils.test.StringUtilsTest


testRowExistsInTable() - Method in class uk.ac.standrews.utils.test.database.TableReaderTest


testSerialNumericalEnding() - Method in class uk.ac.standrews.utils.test.StringUtilsTest


testSeries() - Method in class uk.ac.standrews.utils.test.UtilsTest


testSetFromList() - Method in class uk.ac.standrews.utils.test.ListUtilsTest


testShowAMatrixOfDoubles() - Method in class uk.ac.standrews.utils.test.stats.UniformRandomUtilsTest
:   To check they are not always the same...

testSimpleElement() - Method in class uk.ac.standrews.utils.test.XmlNodeTest


testStringContainsPositiveInteger() - Method in class uk.ac.standrews.utils.test.StringUtilsTest


testStringIsNullOrEmpty() - Method in class uk.ac.standrews.utils.test.ListUtilsTest


testStringIsNullOrEmpty() - Method in class uk.ac.standrews.utils.test.StringUtilsTest


testStringMatchesRegExp() - Method in class uk.ac.standrews.utils.test.RegExpUtilsTest


testStripHtml() - Method in class uk.ac.standrews.utils.test.RegExpUtilsTest


testSubstituteMatchedString() - Method in class uk.ac.standrews.utils.test.RegExpUtilsTest


testSurroundWith() - Method in class uk.ac.standrews.utils.test.database.SqlUtilsTest


testTableColumnsAreSorted() - Method in class uk.ac.standrews.utils.test.database.TableTest


testTableExistsInDb() - Method in class uk.ac.standrews.utils.test.database.DbReaderTest


testToString() - Method in class uk.ac.standrews.utils.test.maputils.MapOfMapsTest


testToString() - Method in class uk.ac.standrews.utils.test.XmlNodeTest


testTrimList() - Method in class uk.ac.standrews.utils.test.ListUtilsTest


testTruncatedPareto() - Method in class uk.ac.standrews.utils.test.stats.TruncatedParetoTest


testUniqueValues() - Method in class uk.ac.standrews.utils.test.maputils.MapOfMapsTest


TEXT\_SQL\_DATATYPE - Static variable in class uk.ac.standrews.utils.main.database.DbUtils


THRESHOLD\_100 - Static variable in class uk.ac.standrews.utils.test.stats.TruncatedParetoTest


tidyDOM(InputStream) - Static method in class uk.ac.standrews.utils.main.webutils.XMLUtils
:   JTidy is designed to read-in typical-quality (that is to say, bad) HTML
    and output something cleaner (you have a choice of options), and also
    provides a DOM interface for traversing HTML documents that can be fed to
    an XML parser.

TIMESTAMP - Static variable in class uk.ac.standrews.utils.main.database.DbUtils


TIMESTAMP\_DOWNLOADED - Static variable in class uk.ac.standrews.utils.main.database.DbUtils


TIMESTAMP\_SQL\_DATATYPE - Static variable in class uk.ac.standrews.utils.main.database.DbUtils


timestampedComment(String) - Static method in class uk.ac.standrews.utils.main.TimeUtils


TimeUtils - Class in uk.ac.standrews.utils.main
:   Utilities to manipulate time objects, create timestamps etc.

TimeUtils() - Constructor for class uk.ac.standrews.utils.main.TimeUtils


TimeUtilsTest - Class in uk.ac.standrews.utils.test
:   Class

TimeUtilsTest() - Constructor for class uk.ac.standrews.utils.test.TimeUtilsTest


toCSV() - Method in class uk.ac.standrews.utils.main.maputils.OneToManyMap
:   Get a list of comma separated [key, value] couples.

toReadableString() - Method in class uk.ac.standrews.utils.main.collectionutils.setutils.Set


toReadableString() - Method in class uk.ac.standrews.utils.main.setutils.Set


toString() - Method in class cern.jet.random.Pareto
:   Returns a String representation of the receiver.

toString() - Method in class uk.ac.standrews.utils.main.collectionutils.setutils.Set


toString() - Method in class uk.ac.standrews.utils.main.database.TableRow
:   Returns a comma separated string of the values

toString() - Method in class uk.ac.standrews.utils.main.diff.Difference
:   Returns a string representation of this difference.

toString() - Method in class uk.ac.standrews.utils.main.guiutils.CommandLineMenu
:   "-n  The file containing the network e.g.

toString() - Method in class uk.ac.standrews.utils.main.guiutils.CommandOption


toString() - Method in class uk.ac.standrews.utils.main.maputils.MapOfMaps


toString() - Method in class uk.ac.standrews.utils.main.maputils.OneToManyMap


toString() - Method in class uk.ac.standrews.utils.main.setutils.Set


toString() - Method in class uk.ac.standrews.utils.main.webutils.simpledomparser.XmlNode
:   Full string representation of node and all child nodes.

treeMapsHaveEqualValues(TreeMap<Integer, Character>, TreeMap<Integer, Character>) - Static method in class uk.ac.standrews.utils.main.maputils.MapUtils
:   Check if two treeMaps have the same collection of values

trimList(Vector<String>) - Static method in class uk.ac.standrews.utils.main.collectionutils.VectorUtils
:   trim all trailing white spaces from strings in a list (beware: null
    elements become empty strings)

TruncatedPareto - Class in uk.ac.standrews.utils.main.stats
:   A utility class to extract from a truncated Pareto (power-law, long-tail)
    distribution.

TruncatedPareto(double, double, int, int) - Constructor for class uk.ac.standrews.utils.main.stats.TruncatedPareto


TruncatedParetoTest - Class in uk.ac.standrews.utils.test.stats
:   Class

TruncatedParetoTest() - Constructor for class uk.ac.standrews.utils.test.stats.TruncatedParetoTest

A B C D E F G H I J K L M N O P Q R S T U V W X Y

- Overview
- Package
- Class
- Use
- Tree
- Deprecated
- Index
- Help

- Prev Letter
- Next Letter

- Frames
- No Frames

- All Classes
